# Supplementary material for: Single-Cell RNA-Seq Analysis Reveals the Acquisition of Cancer Stem Cell Traits and Increase of Cell–Cell Signaling during EMT Progression
Source: Cancers (Basel). 2021 Nov 16;13(22):5726. doi: 10.3390/cancers13225726 (PMC8616061; doi:10.3390/cancers13225726)
Supplement: Supplementary file 1 [file cancers-13-05726-s001.zip › cancers-1403366-supplementary.pdf]

Single cell RNA-seq analysis reveals acquisition of cancer stem cell traits and increase of cell-cell signaling during EMT progression

Federico Bocci[1,2], Peijie Zhou[1], Qing Nie[1,2,*]

[1]Department of Mathematics, University of California, Irvine, CA, United States; [2]The NSF-Simons Center for Multiscale Cell Fate Research, University of California, Irvine, CA, United States

*Author for correspondence: qnie@uci.edu

Supplementary Material

Supplementary Figures

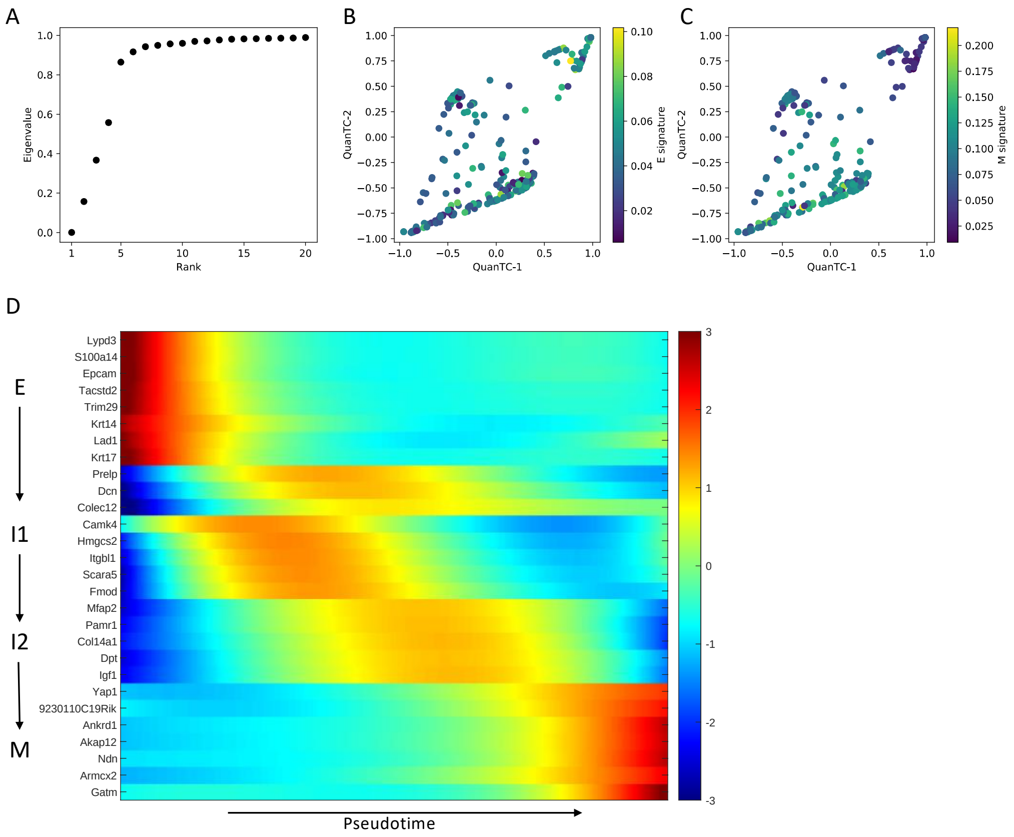

Supplementary Figure S1. **Clustering and analysis of SCC cells. (A)** Eigenvalues of the similarity matrix ranked in ascending order. **(B-C)** Epithelial and Mesenchymal signatures of cells in low-dimensional QuanTC projection space. **(D)** Top marker and transition genes identified by QuanTC along the most probable transition path (i.e., the path indicated by black arrows in Fig. 1B).

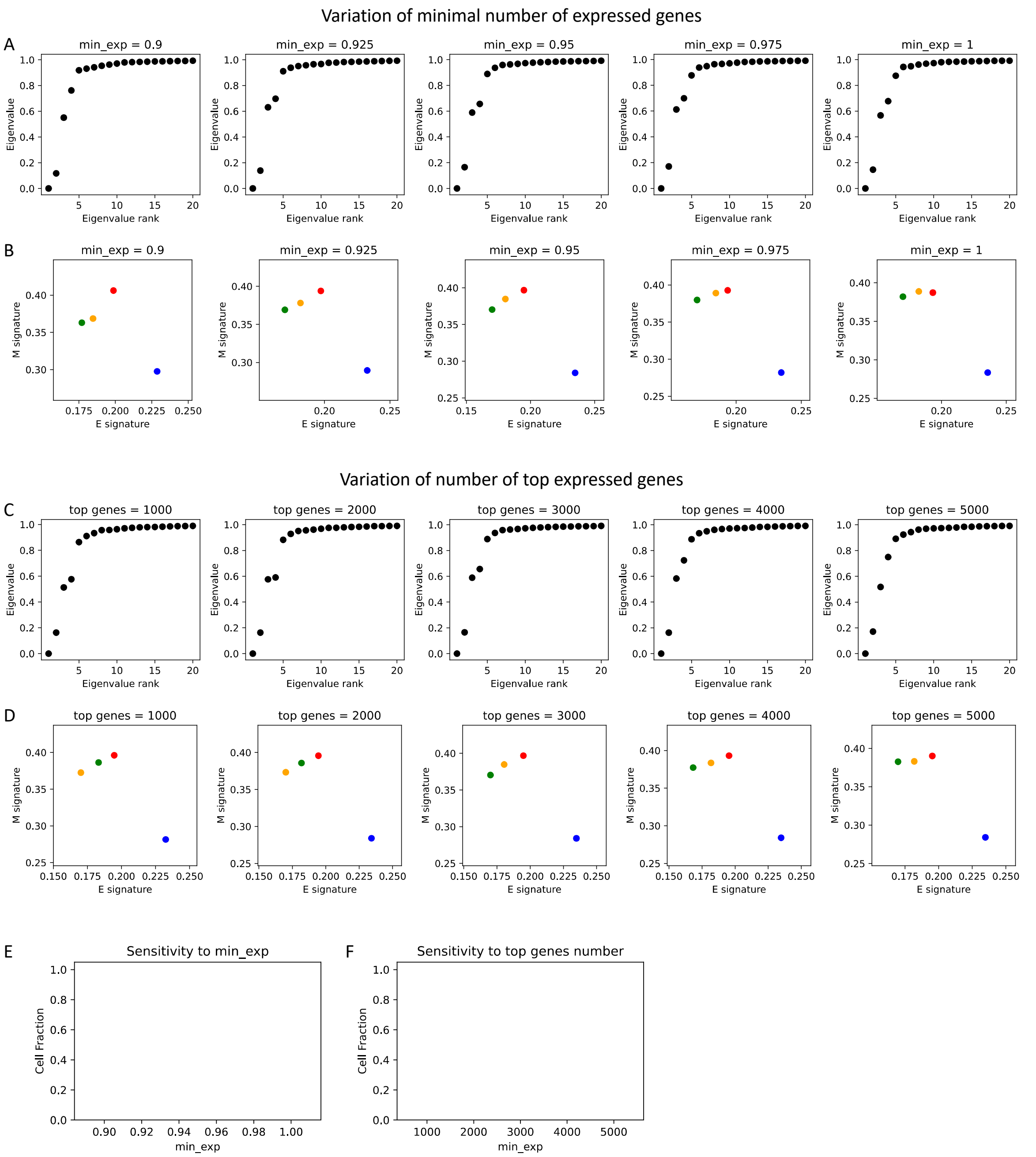

Supplementary Figure S2. **Robustness of SCC clustering. (A)** Eigenvalues of the similarity matrix ranked in ascending order for increasing values of min_exp, the minimal fraction of expressed genes in QuanTC preprocessing (same as in Fig. S1A, default value = 0.95 used in Fig. 1). **(B)** Projection of clusters in the E-M signature space for increasing values of min_exp (same as in Fig. 1B). **(C)** Eigenvalues of the similarity matrix for increasing values of ngenes, the number of top expressed genes to selected for QuanTC analysis (default value = 3000). **(D)** Projection of clusters in the E-M signature space for increasing values of ngenes. **(E)** Fractions of cells in the E, I1, I2, and M clusters for increasing values of min_exp. **(F)** Same as (E) for increasing values of ngenes.

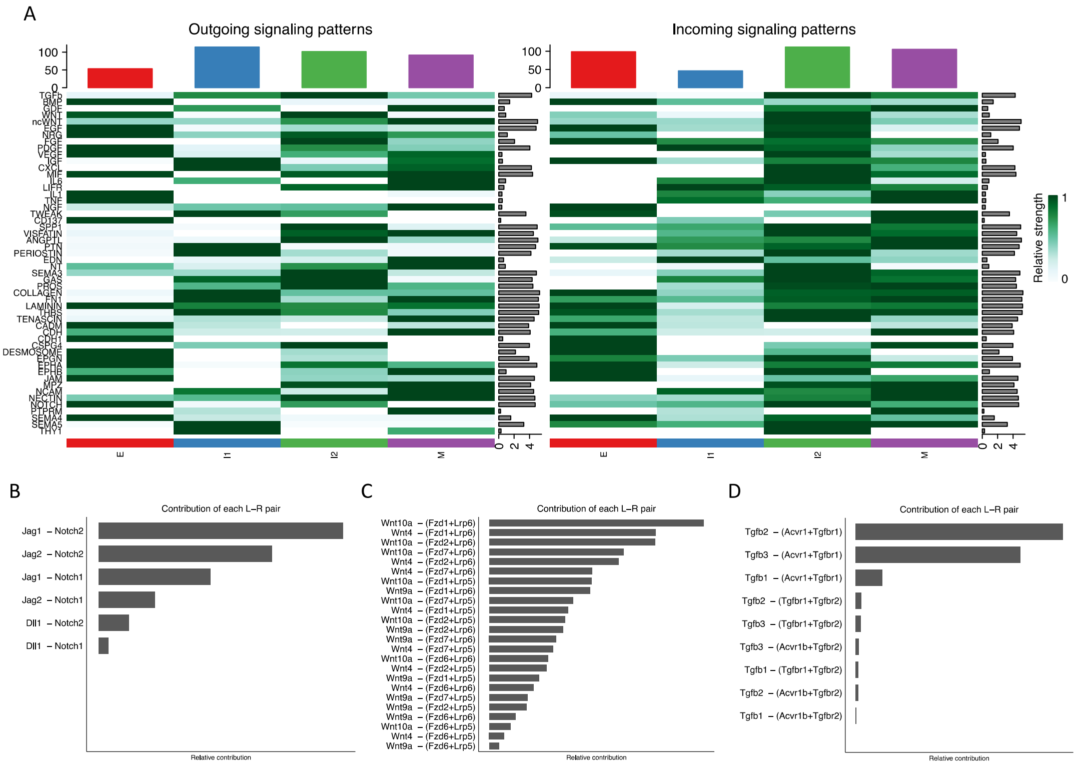

Supplementary Figure S3. **Cellchat analysis of SCC cells. (A)** Quantification of Outgoing and Incoming signaling patterns quantified by CellChat. Top bar plots summarize the Outgoing and Incoming signaling of each cluster (all pathways considered), while the rightmost bar plots quantify the overall strength of the specific pathways (all clusters considered). **(B-C-D)** Breakdown of the specific ligand-receptor pairs that contribute to Notch (B), WNT (C) and TGF-beta signaling (D).

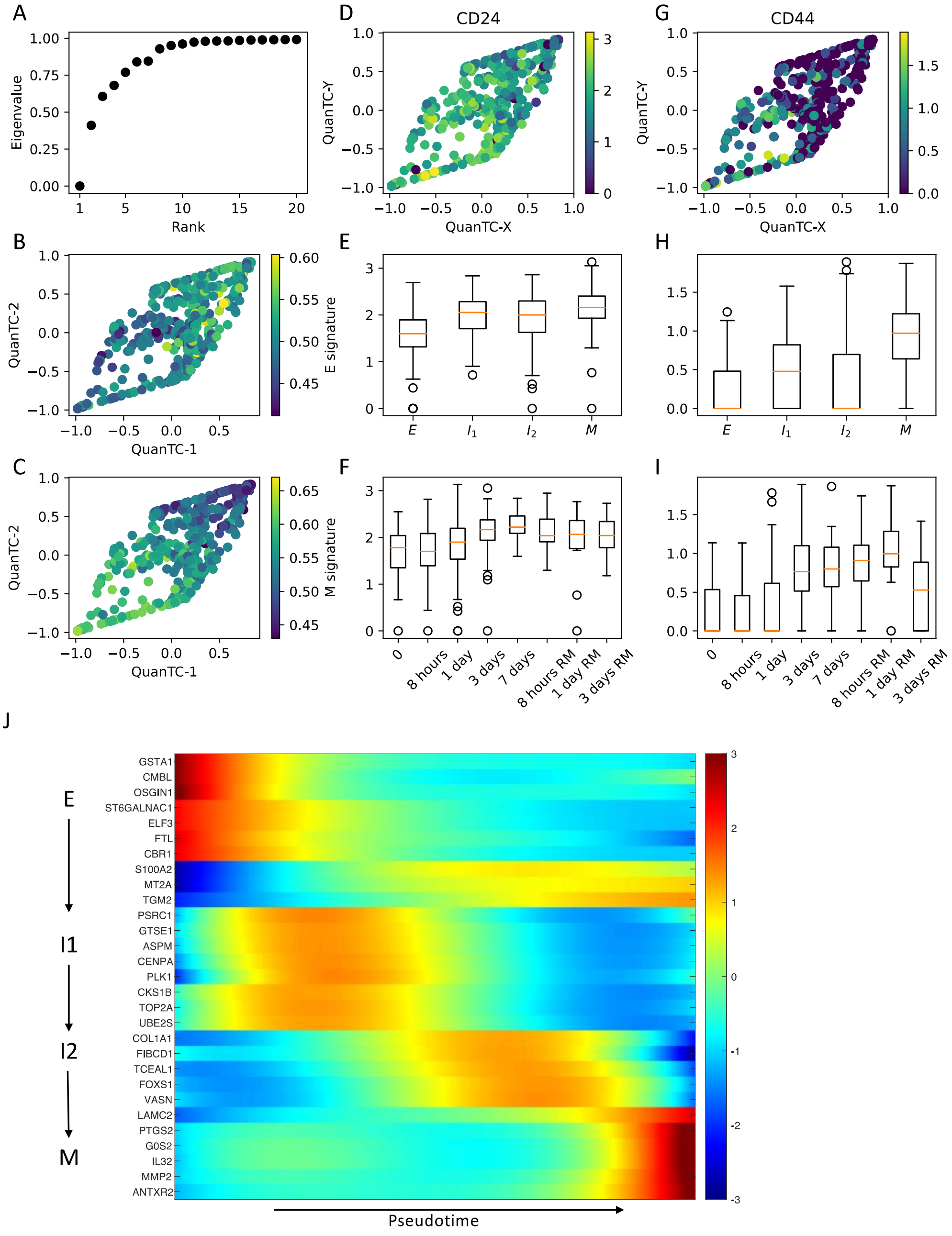

Supplementary Figure S4. **Clustering and analysis of OVCA420 cells under TGFB. (A)** Eigenvalues of the similarity matrix ranked in ascending order. **(B-C)** Epithelial and Mesenchymal signatures of cells in low-dimensional QuanTC projection space. **(D)** Scatterplot of CD24 in QuanTC space. **(E)** Boxplot of CD24 expression by cluster. **(F)** Boxplot of CD24 expression by anatomical location. **(G-H-I)** Same as (D-E-F) for CD44. **(J)** Top marker and transition genes identified by QuanTC along the most probable transition path (i.e., the path indicated by black arrows in Fig. 2B).

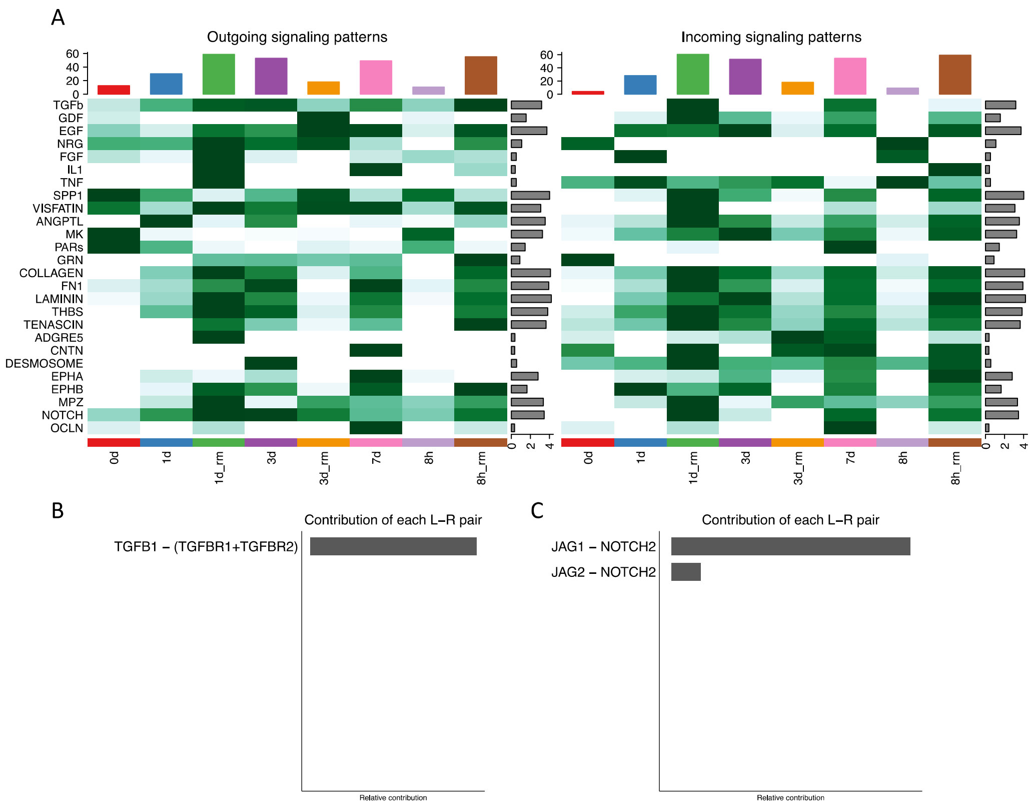

Supplementary Figure S5. **Cellchat analysis of OVCA420 cells under TGFB. (A)** Quantification of Outgoing and Incoming signaling patterns quantified by CellChat. **(B)** Breakdown of the specific ligand-receptor pairs that contribute to TGF-beta signaling. **(C)** Same as (B) for Notch signaling.

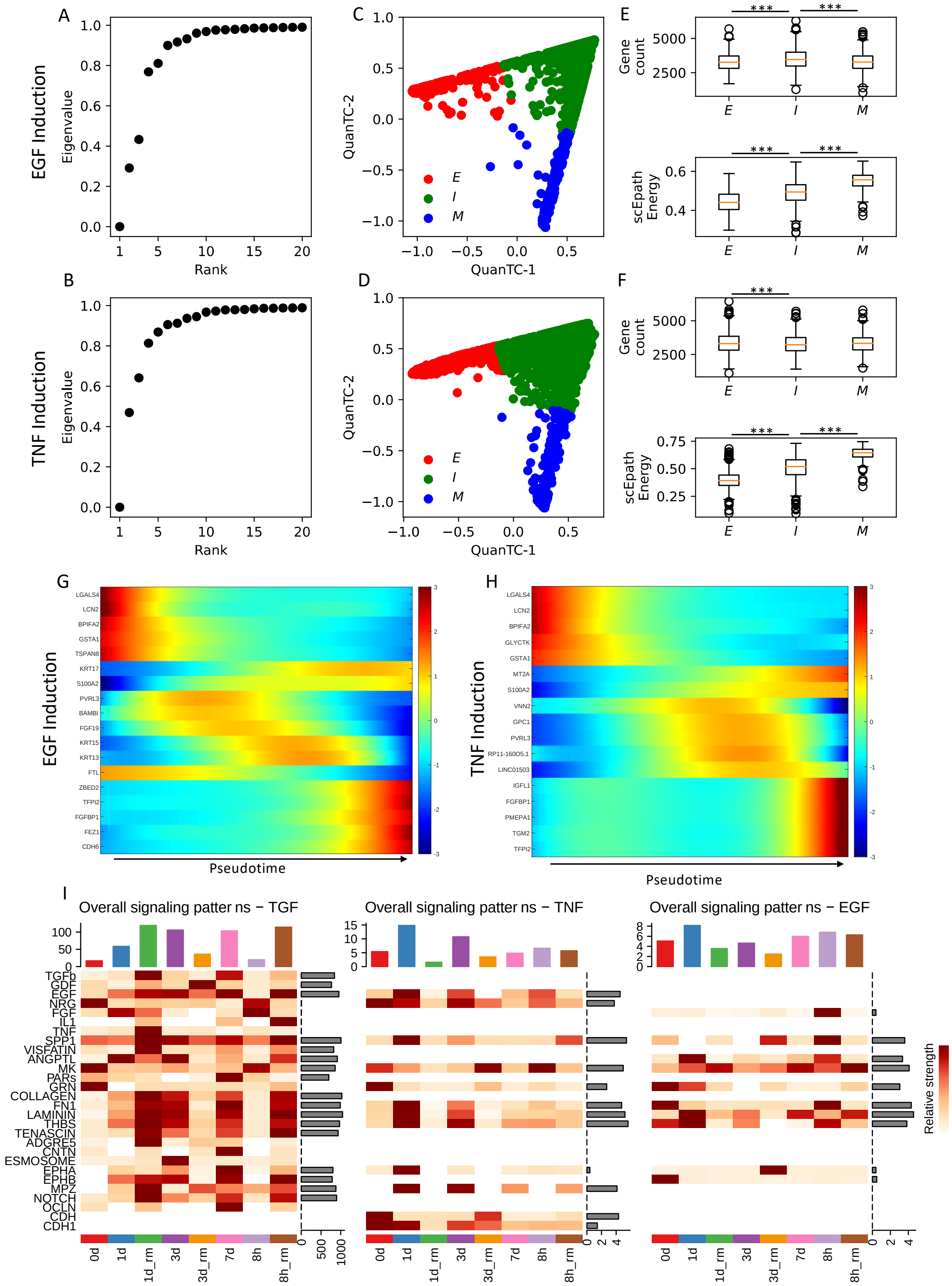

Supplementary Figure S6. **Comparison between EMT inducers in OVCA420 cells. (A)** Eigenvalues of the similarity matrix ranked in ascending order for OVCA420 cells under EGF induction. **(C)** Low-dimensional representation of cells in the OVCA420 dataset under EGF induction. **(E)** Transcriptional diversity and developmental energy for OVCA420 cells under EGF induction. **(B-D-F)** Same as (A-C-E) for TNF induction. **(G-H)** Top marker and transition genes identified by QuanTC along the most probable transition path for EGF-driven EMT (G) and TNF-driven EMT (H). **(I)** Comparison of overall signaling pathway strength between TGF-beta induction, EGF induction and TNF induction computed with CellChat.

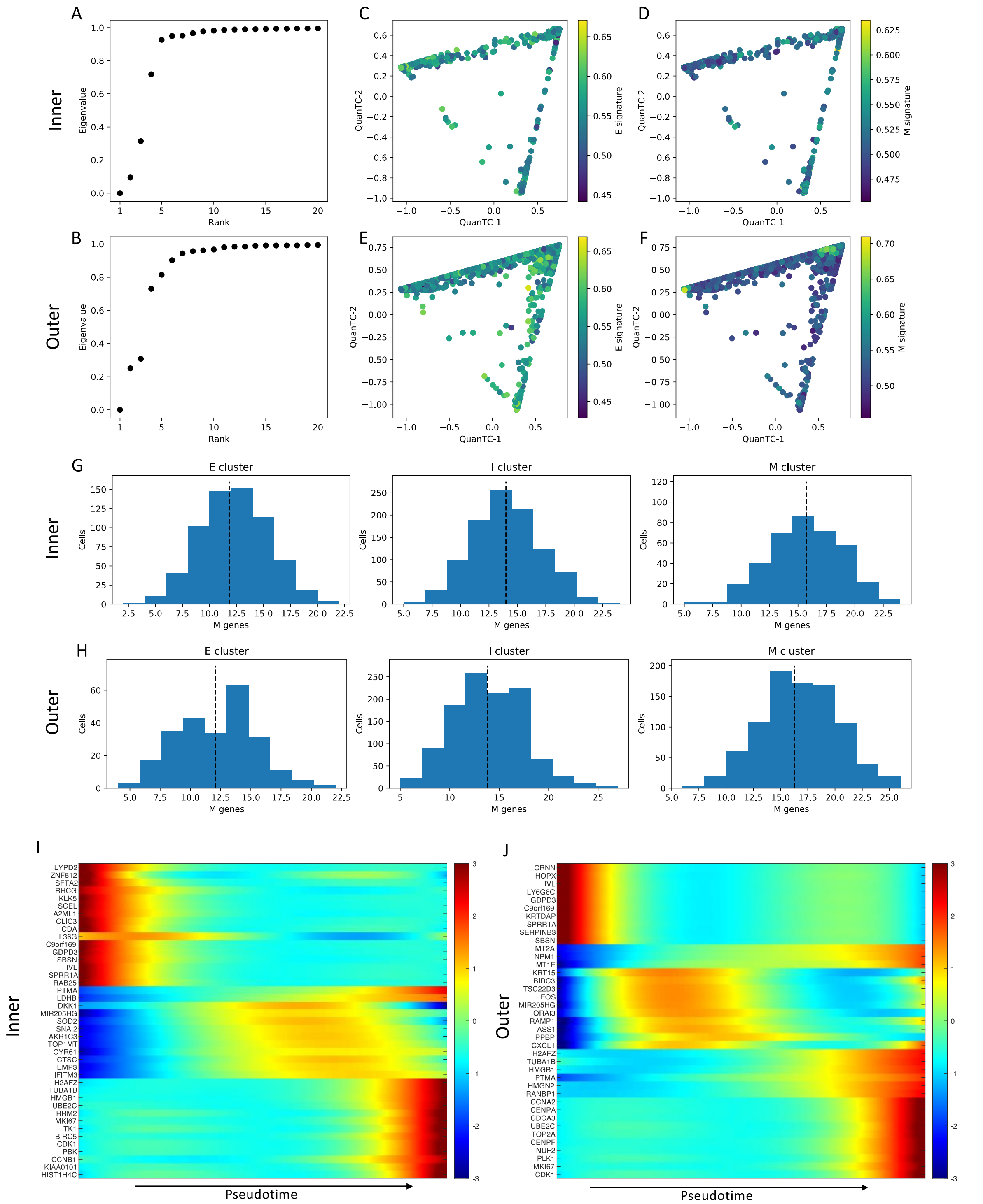

Supplementary Figure S7. **Clustering and analysis of MCF10A cells. (A-B)** Eigenvalues of the similarity matrix ranked in ascending order for the inner (A) and outer (B) cell populations. **(C-D-E-F)** Epithelial and Mesenchymal signatures of cells in low-dimensional QuanTC projection space for the inner (C-D) and outer (E-F) cell populations. **(G-H)** Histograms showing the number of mesenchymal genes (i.e., up-regulated EMT genes) expressed in MCF10A in the E, I, and M clusters in the Inner (G) and Outer (H) datasets, respectively. **(I-J)** Top marker and transition genes identified by QuanTC along the most probable transition path in the inner (I) and outer (J) cell populations.

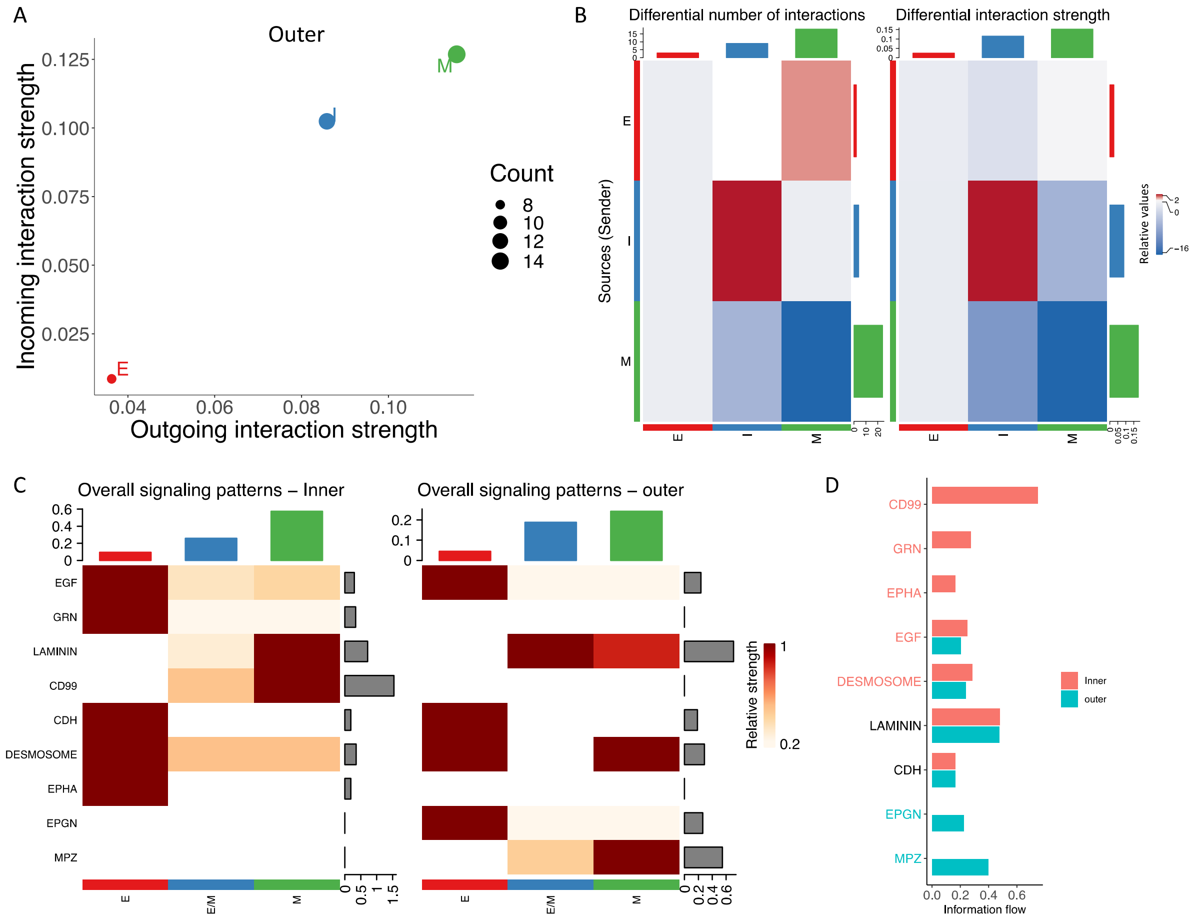

Supplementary Figure S8. **Cellchat analysis of MCF10A cells. (A)** Low-dimensional projection of cell clusters from the Outer dataset in an incoming/outgoing signaling strength space computed with CellChat. **(B)** Heatmap quantifying the fold-change in pairwise cluster-cluster signaling between the Inner and Outer cell populations. Blue coloring indicates that the signaling is stronger in the Inner population, while red coloring indicates stronger signaling in the Outer population. **(C)** Comparison of signaling patterns between Inner and Outer cell populations. Top bar plots summarize the signaling strength of each cluster (all pathways considered), while the rightmost bar plots quantify the overall strength of the specific pathways (all clusters considered). **(D)** Information flow of highly expressed signaling pathways in the Inner (pink) and Outer (cyan) cell populations.

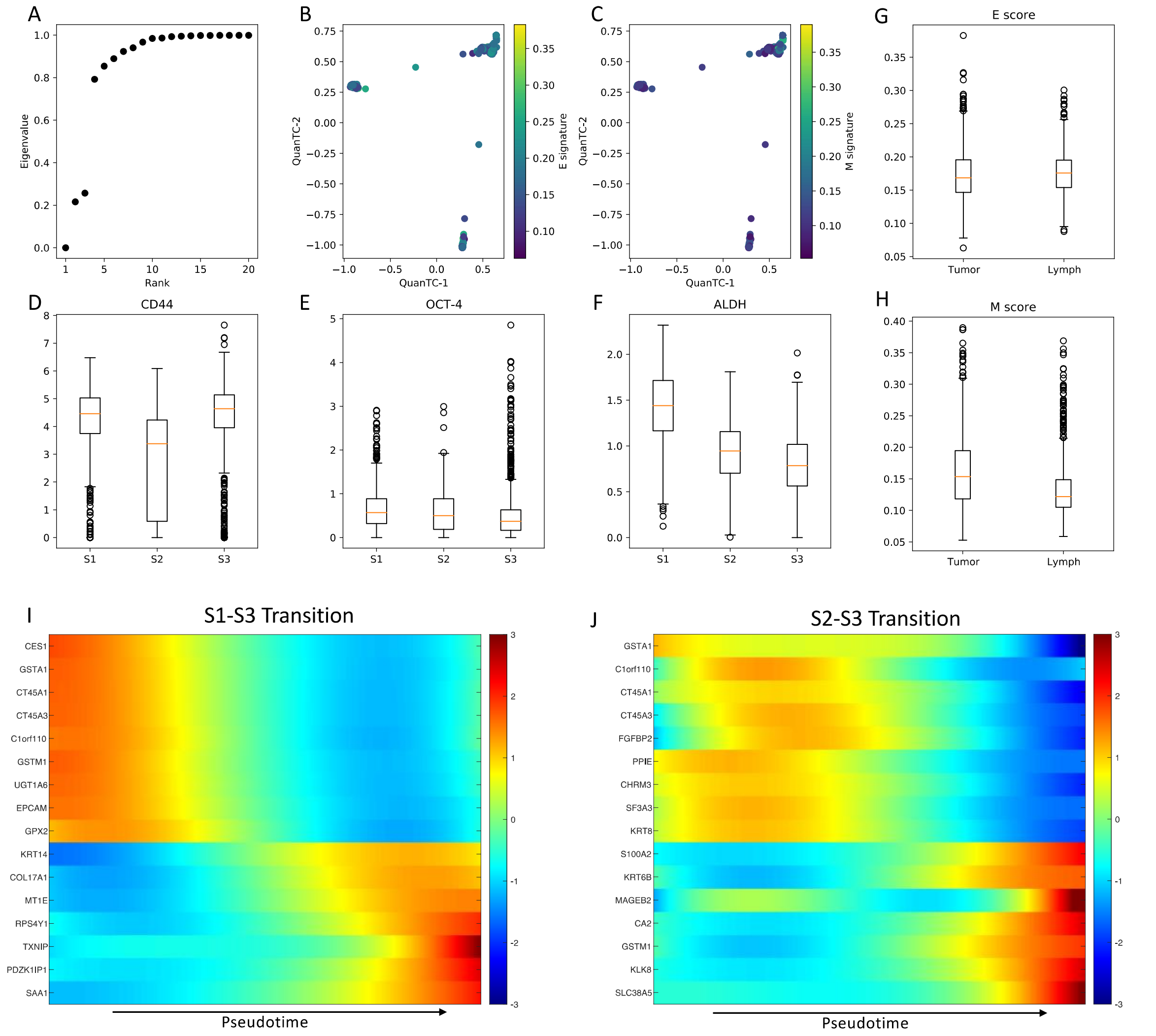

Supplementary Figure S9. **Clustering and analysis of HNSCC cells. (A)** Eigenvalues of the similarity matrix ranked in ascending order for the NHSCC dataset. **(B-C)** Epithelial and Mesenchymal signatures of cells in low-dimensional QuanTC projection space. **(D-E-F)** Boxplot of CD44, OCT-4, and ALDH1 expression in the three HNSCC clusters. **(G-H)** Boxplot of E and scores in tumor vs. lymph node metastasis. **(I-J)** Top marker and transition genes identified by QuanTC along S1-S3 (I) and the S2-S3 (J) transition paths. The S3-S2 transition path exhibits the same heatmap of panel (H) with inverted pseudotime axis.

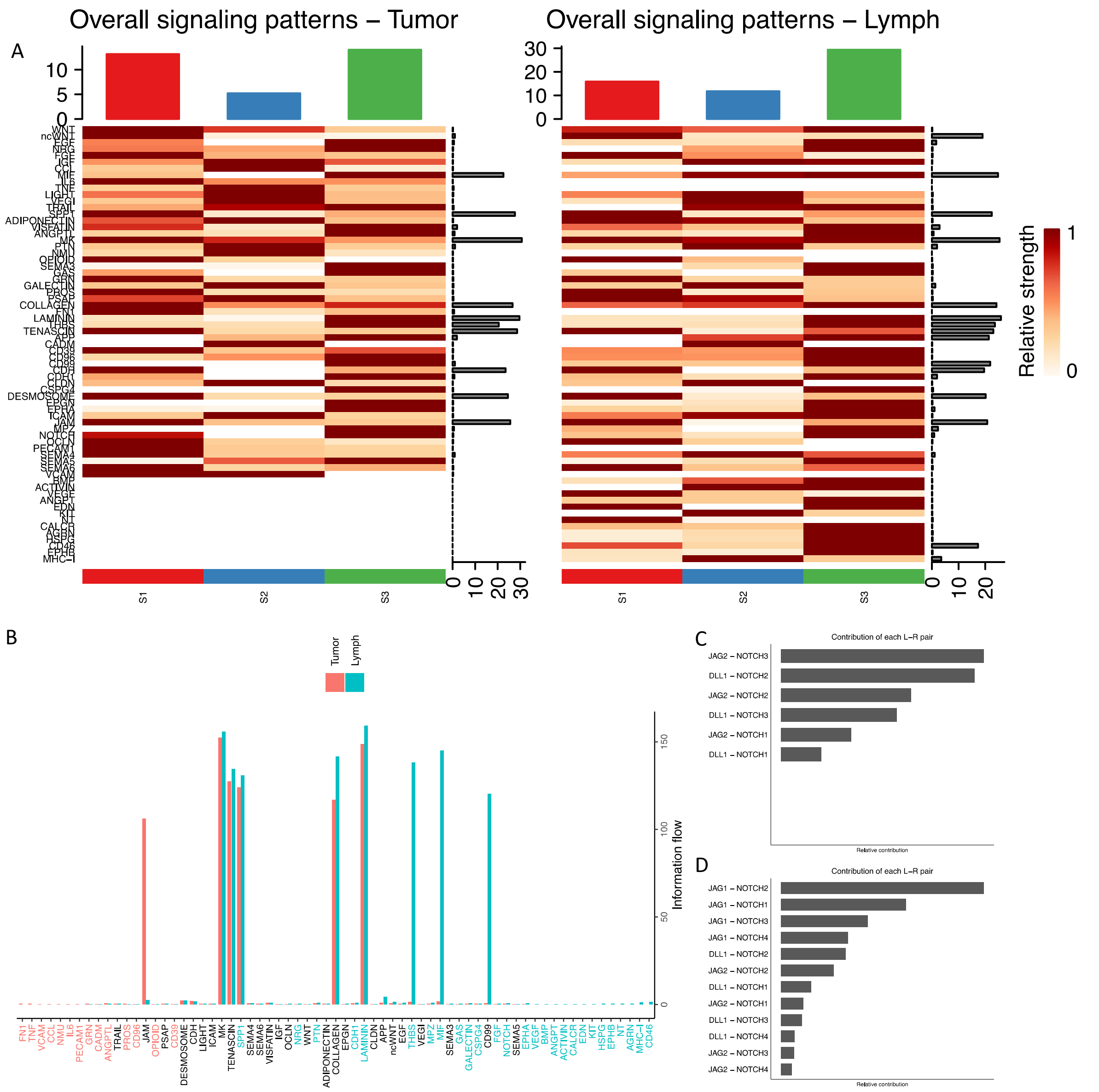

Supplementary Figure S10. **Cellchat analysis of HNSCC cells. (A)** Comparison of signaling patterns between Tumor and Lymph node cells. Top bar plots summarize the signaling strength of each cluster (all pathways considered), while the rightmost bar plots quantify the overall strength of the specific pathways (all clusters considered). **(B)** Information flow of highly expressed signaling pathways in the Tumor (pink) and Lymph nodes (cyan). **(C-D)** Comparison of pairwise ligand-receptor contribution to Notch signaling in tumor (C) vs lymph nodes (D).

Supplementary Table S1. List of up-regulated and down-regulated EMT genes (separate file).

E genes	M genes
ADORA2B	ABCA1
ALDH1A3	ACTN1
ALDH3A2	JAG1
ANK3	ALOX5AP
BIRC3	APBB2
AQP3	ARNTL
AREG	BMP1
CFB	BMP2
DST	BMPR2
CD9	BPGM
CDH1	CALD1
CEBPD	RUNX2
CP	CD59
CXADR	CDH2
CYB5A	CDH11
CYP1B1	COL1A1
DEFB1	COL3A1
SERPINB1	COL4A1
ELF3	COL4A2
EMP1	COL5A1
EPAS1	COL5A2
ERBB3	COL6A3
EREG	COL7A1
GLDC	VCAN
CFH	CTGF
FOXA2	SLC26A2
HPGD	ELK3
IMPA2	EML1
INHBB	EPHB2
JAG2	ETS2
KRT15	FBN1
KRT19	FOXD1
LAMA5	FN1
LCN2	GNG11
ABLIM1	GRB10
LY6E	HMOX1
TACSTD1	HRH1
MBP	IGFBP5
KITLG	IGFBP7
MITF	IL11
MMP7	INHBA

MUC1	ITGA5
CEACAM6	ITGB3
PDK4	JARID2
ATP8B1	JUN
PKP2	JUNB
PLS1	KCNJ15
PPL	KCNMA1
RARRES3	LAMC2
S100P	LOX
SCNN1A	LUM
SLPI	SMAD7
SORD	MAF
SOX2	MATN3
SULT1A1	MFAP2
TFAP2A	MMP1
NR2F2	MMP2
TNFAIP2	MMP9
TPD52L1	MMP10
ALDH5A1	MN1
SDPR	GADD45B
MAP7	MYO10
SLC16A7	NCF2
ARHGAP29	NEDD9
TJP2	NKX3-1
GDF15	NT5E
HS3ST1	SERPINE1
FGFBP1	PDGFA
TSPAN1	PFTK1
MPZL2	SERPINE2
SPRY1	PIK3CD
CITED2	PLAUR
VAV3	PODXL
AGR2	HTRA1
SLC27A2	PSMD2
TBC1D8	PTHLH
HRASLS3	PTPRK
PEG10	RALA
KIAA0182	RGS4
SYNE2	SCG5
NUP210	SKIL
RAB38	SLCO2A1
RAB26	SLC22A4
METTL7A	SLN
EHF	SNAI2

SMPDL3B	SPARC
SLCO4A1	SPOCK1
HOOK1	STC1
GULP1	TAGLN
LSR	TCF4
FLJ20273	TBX3
EPB41L4B	TGFB1
MANSC1	TGFB1I1
RBM35A	TGFBI
PPP1R9A	TGM2
GOLSYN	THBS1
GPRC5C	TIMP2
MYO5C	TNFAIP6
RAB25	TNS1
MTUS1	TPM1
SQRDL	TPM4
DEPDC6	TUBA4A
C1ORF116	TUFT1
FA2H	VEGFC
C1ORF115	VIM
GRTP1	WNT5A
ERMP1	TUBA1A
TMEM30B	SCG2
	TFPI2
	ADAM12
	HMGA2
	SRPX
	TAGLN2
	TPST2
	TPST1
	BHLHB2
	KLF7
	PEA15
	ADAM19
	INPP4B
	SPHK1
	AP1S2
	CRLF1
	PDLIM7
	PSCD1
	C5ORF13
	TP53I3
	MICAL2
	DOCK4

	NUAK1
	MRC2
	GFPT2
	HS3ST3B1
	HS3ST3A1
	AKT3
	DHRS2
	FSTL3
	DLC1
	MYL9
	SEMA3C
	POSTN
	MAGED2
	FERMT2
	GLIPR1
	KDELR3
	HSF2BP
	PTPN21
	ADAMTS6
	DUSP10
	ZNF365
	DAAM1
	PALLD
	TMCC1
	KIAA0692
	RFTN1
	SACS
	PLEK2
	GREM1
	LOH3CR2A
	DSE
	LMCD1
	CHST11
	GAL
	ANGPTL4
	C4ORF18
	DACT1
	CCDC99
	PID1
	LARP6
	GALNT10
	CHRNA9
	FLJ10357
	ARFGAP1

	PDGFC
	NRIP3
	PMEPA1
	CXCR7
	XYLT1
	SMURF2
	C3ORF52
	FLJ14213
	FHOD3
	WNT5B
	LBH
	MAP1LC3B
	DIXDC1
	FAM114A1
	MBOAT2
	SIK1
	C6ORF145
	AMIGO2
	VGLL3
	SRRD

Path	Percentage of cells involved
**SCC**	
E-I1-I2-M	84.2%
E-I1-M	65.6%
E-I2-I1-M	50.7%
E-I2-M	26.0%
E-M	13.8%
**OVCA420 - TGFB**	
E-I1-I2-M	78.3%
E-I2-I1-M	50.6%
E-I1-M	45.9%
E-I2-M	35.5%
E-M	8.8%
**OVCA420 - EGF**	
E-I-M	83.1%
E-M	21.6%
**OVCA420 - TNF**	
E-I-M	95.1%
E-M	5.8%
**MCF10A - Inner**	
E-I-M	95.4%
E-M	14.3%
**MCF10A - Outer**	
E-I-M	96.6%
E-M	17.1%
**HNSCC**	
S1-S3	81.5%

S2-S3	18.4%
S3-S2	18.4%

Supplementary Table S2. EMT paths identified by QuanTC. Note: cells can be members of more than one pathway; therefore, the percentages do not sum to 100%.
